# Gastric Electrical Stimulation for Treatment of Refractory Gastroparesis: the Current Approach to Management

**DOI:** 10.1007/s11894-020-00803-0

**Published:** 2021-01-22

**Authors:** Aaron Shanker, Mohammad Bashashati, Ali Rezaie

**Affiliations:** 1 Department of Internal Medicine, Texas Tech University Health Sciences Center, El Paso, TX USA; 2 Division of Gastroenterology, Department of Internal Medicine, Texas Tech University Health Sciences Center, El Paso, TX USA; 3 GI Motility Program, Cedars-Sinai Medical Center, Los Angeles, CA USA

**Keywords:** Gastroparesis, Gastric electrical stimulation, Vomiting, Nausea, Abdominal pain, Motility disorders

## Abstract

**Purpose of Review:**

Gastroparesis is one of the more challenging entities in the landscape of gastroenterology, posing difficulties for both patients and physicians with regard to effective management and therapies. In this article, we reviewed various gastroparesis treatment options, with an emphasis on gastric electrical stimulation (GES).

**Recent Findings:**

GES has demonstrated a significant reduction of cardinal symptoms in refractory gastroparetic patients, particularly nausea and vomiting, across multiple studies. However, GES has not been shown to conclusively decrease gastric emptying time in these patients. Such finding has led the investigators to analyze the impact of combining GES with pyloroplasty. While this treatment pathway is nascent, its results thus far reveal an amplified improvement of gastroparesis symptomatology in addition to significant reduction of gastric transit, compared to GES by itself.

**Summary:**

Limited treatment choices are available for refractory gastroparesis. Combining GES with pyloroplasty holds promise but requires further assessment in large-scale trials to fully evaluate the risks and benefits.

## Introduction

Gastroparesis is characterized as a syndrome of delayed gastric emptying with associated cardinal symptoms such as early satiety, postprandial fullness, nausea, vomiting, bloating, and upper abdominal pain. This disorder is marked by an absence of mechanical gastric outlet obstruction [1]. The age-adjusted prevalence of gastroparesis per 100,000 persons in 2017 was 9.6 for men (95% CI, 1.8–17.4) and 37.8 for women (95% CI, 23.3–52.4). The predominance of gastroparesis among female patients compared to male patients is a finding that has been reflected in multiple studies [2].

Gastroparesis poses a major burden to the economy, manifesting its effects via clinic visits, emergency room visits, hospitalizations, and treatments. Furthermore, gastroparesis patients may experience employment, education, and disability assistance difficulties. These factors should be scrutinized in tandem with the mental, emotional, and psychological tolls of this disease, adversely impacting a patient’s quality of life [3]. From 1997 to 2013, admissions due to gastroparesis increased by 300%, and while a decrease in average length of hospital stay was exhibited, this occurred in the setting of significantly increased cost of each hospitalization [4, 5]. From ethnicity perspectives, from 1997 to 2013, black gastroparetic patients witnessed a 4.5-fold increase in hospital discharges, compared to 5.5-fold increase among Hispanic gastroparetic patients and 3-fold increase among white gastroparetic patients [4].

The precise mechanism of gastroparesis’ underlying pathophysiology has not been clearly elucidated yet. The most frequently cited causes of gastroparesis are diabetes and idiopathic and postsurgical states [6]. Other less commonly implicated causes include medications (especially those which reduce gastric transit time) such as opioids, antibiotics, antiarrhythmics, and anticonvulsants; neurologic disorders such as Parkinson’s disease, amyloidosis, and dysautonomia; post-viral infections such as norovirus, Epstein Barr virus, cytomegalovirus, and herpes virus; and connective tissue disorders such as scleroderma and systemic lupus erythematosus [7, 8].

Treatment options at this juncture (which will be discussed later) are limited in scope and may “not adequately address clinical need” [6]. The aim of this paper is to capture the disease process of gastroparesis on a micro- and macro-level, analyze the impact of gastric electrical neurostimulation as a therapeutic modality, and forecast the potential of combined gastric electrical neurostimulation with surgical pyloroplasty to achieve sustained improved outcomes.

## Pathophysiology of Gastroparesis

While there is no single established mechanism of action that defines the pathophysiology of gastroparesis, much attention and research have been recently devoted to the role of interstitial cells of Cajal (ICC) in the disease’s etiology [9]. ICCs are recognized as the electrical pacemaker cells of the stomach. Their ability to generate slow waves is an essential component of the smooth muscle contractility of the gastrointestinal system [10].

Gastric electrical rhythms (slow waves) are generated by ICCs and propagate around and down the stomach toward the antrum. These waves are essential for coordinating gastric peristaltic contractions which triturate and empty ingested foods. The normal slow waves are 3 cycles per minute (cpm) [11, 12]. In gastroparesis, there is depletion of ICCs throughout the stomach, more prominently in the pylorus. Moreover, studies have shown increased smooth muscle fibrosis and decreased enteric neurons in gastroparesis [9, 13]. ICC depletion can affect slow-wave cycles and induce gastric dysrhythmias, including tachygastrias [14, 15]. Gastroparetic patients have a greater total symptom score (TSS) compared with gastroparesis-like patients. In gastroparetic patients, those with pyloric ICC loss had a greater TSS compared with those with normal pyloric ICC levels [13, 16].

Gastric dysrhythmias are common in gastroparesis and manifest as tachygastrias (3.75–10 cpm), bradygastrias (1.0–2.5 cpm), and arrhythmias. Remarkably, gastric dysrhythmias are more common during nausea [15, 17]. Therefore, tuning of the intrinsic gastric electrical activity with extrinsic stimulation would have therapeutic potentials in these patients and notably should be more effective for nausea [18].

## Disease Severity

To better gauge a gastroparetic patient’s clinical condition and response to management and treatment, the American Neurogastroenterology and Motility Society developed the Gastroparesis Cardinal Symptom Index Daily Diary (ANMS GCSI-DD). Patients are asked to complete the questionnaire on a daily basis at the same time every evening. The assessment tool is built as a 5-point Likert scale to assess the degree of intensity of the following five symptoms (bloating, nausea, early satiety, postprandial fullness, and upper abdominal pain). In addition, patients are asked to document the amount of vomiting episodes they have had in the last 24 h. Lastly, patients are asked to evaluate the severity of their symptoms over the past 24 h. The daily score is then computed with possible scores ranging from 0 to 4. In ascending order, these scores correspond to a disease severity of none, mild, moderate, severe, or very severe. Thus, “high scores on the ANMS GSCI-DD reflect greater symptom severity” [19].

More practically, gastroparesis is categorized as (1) mild with no daily symptoms, no hospitalizations, and no impact on work and family functioning; (2) moderate gastroparesis with daily symptoms which are not continuous and cause occasional hospitalization and interfere with work and family functioning; and (3) severe gastroparesis with daily, continuous symptoms, multiple ED visits/hospitalizations, and not able to work and function [20]. While it is expected that gastric emptying rate may predict disease severity and response to therapy, the findings on the association between these two parameters are controversial [21].

## Treatment Options in Gastroparesis

### Non-invasive Treatment Options

In regard to therapy, treatment options are multifactorial and multidisciplinary. Lifestyle modifications, such as dietary changes, play a role in symptom control. Emphasis is placed on multiple small meals throughout the day. Patients may target consuming four to six meals at least in 1 day. These meals should be low in fat and fiber content, as those elements may slow gastric transit. In patients dealing with oral intolerance, clinicians may pursue enteral nutrition. Such attempts should begin with a naso-jejunal tube first to evaluate how patients’ symptoms respond with the commencement of feedings. If deemed appropriate, a jejunostomy feeding tube would then be the next step [10]. Of note, enteral nutrition is preferred over total parenteral nutrition (TPN). TPN and its access sites are associated with liver disease and significant risk of infection, particularly in the diabetic population [22]. Because diabetes represents a defining etiology in a significant subset of gastroparesis patients, control of blood sugar is an important component of treatment as well. Hyperglycemia can slow down gastric emptying of both solids and liquids [10, 23]. Poor glycemic control has also been ascribed to feelings of postprandial fullness [24].

Overall, the aim of medical therapy in gastroparesis is to control nausea with antiemetics, control pain with neuromodulating medications such as antidepressants while avoiding narcotics, restore nutrition, electrolytes, and hydration, as well as to coordinate gastric and small bowel motility with prokinetic agents.

Pharmaceutical agents in the management of gastroparesis are limited. The prokinetic metoclopramide (a dopamine receptor antagonist) is the only medication approved by the U.S. Food and Drug Administration (FDA) for the treatment of gastroparesis. However, it carries a US boxed warning to avoid use for longer than 12 weeks duration due to the risk of developing tardive dyskinesia which can be irreversible. Extrapyramidal symptoms, depression, and drug-induced parkinsonism can also be seen with metoclopramide but generally subside with discontinuation of the drug. Neuroleptic malignant syndrome has also been reported. Multiple trials announced the drug improved clinical symptoms and gastric emptying as well [6]. Domperidone is another prokinetic agent with a similar mechanism of action as metoclopramide and equally as effective, but with a lower side effect profile. However, this medication is only available through special FDA programs. QT prolongation is an important potential adverse effect of domperidone, thus necessitating regular EKG monitoring [6]; however, several clinical trials have deemed domperidone an effective and safe medication for gastroparesis [25–27]. Erythromycin, which acts as a motilin receptor agonist, has been shown to improving gastric transit and symptoms, but is associated with diminishing response of the drug in subsequent doses due to downregulation of the motilin receptor [6]. Other medications used in gastroparesis management include antiemetics such as prochlorperazine, promethazine, and ondansetron. Selective 5-HT_4_ receptor agonists such as prucalopride have also shown promise in the management of gastroparesis [28]. While tricyclic antidepressants may be considered in causes of refractory nausea and vomiting, caution must be exercised as some of these drugs may exhibit anticholinergic effects which could work to slow down gastric transit [6]. It is crucial for gastroparetic patients to cease narcotic usage due to their role in decreasing gastric emptying and their implication in symptoms of nausea, vomiting, and abdominal pain [6].

### Minimally Invasive and Invasive Options

Endoscopic interventions such as botulinum toxin injection into the pylorus [29], gastric peroral endoscopic myotomy (G-POEM) of the pylorus [30], and surgical interventions including jejunal tube placement, pyloroplasty, pyloromyotomy, gastric electrical stimulation (GES) implantation, gastric resection, and total gastrectomy are among the treatment options for gastroparesis patients with more severe symptoms [31].

## Gastric Electrical Stimulation

GES is indicated for the treatment of chronic, intractable nausea, and vomiting secondary to diabetic or idiopathic gastroparesis under a humanitarian device exemption (HDE). Implantation of the Enterra gastric electrical stimulator is a surgical procedure. The surgeon may opt for a laparotomy or a less-invasive laparoscopy. The entire apparatus, manufactured by Medtronic, is comprised of two leads, a pulse generator and a programming system [32]. The two neuromuscular leads are placed 1 cm apart from each other within the muscularis propria of the stomach’s greater curvature, at a distance of 10 cm proximal to the pylorus. The pulse generator of the Medtronic Model 4351 has the following stimulation parameters: amplitude: 5 mA, pulse width: 330 μs, cycle: 12 cpm (on time: 0.1 s–14 Hz; off time: 5.0 s). This pulse generator is positioned in the abdominal wall, usually in either the right or left upper quadrant. An external programming system allows for different stimulation parameters. Stimulation via Enterra therapy occurs at high-frequency and low-energy settings. The battery usually lasts 5 to 10 years. Should a battery require replacement, this can be performed without replacing the electrodes [33].

Many researchers have conducted patient trials to evaluate the effects of Enterra GES therapy on patients with gastroparesis. McCallum and his team designed one such study, prospectively investigating 55 patients with refractory diabetic gastroparesis who were selected for Enterra device implantation in a double-blinded and randomized manner encompassing eight centers. At an initial 6-week follow-up period, significant reduction in the patients’ severity and frequency of nausea, vomiting, early satiety, bloating, postprandial fullness, and epigastric pain (documented by patients on a daily basis) were appreciated. This reduction increased for those subjects who were able to complete the 12-month follow-up milestone. The weekly vomiting frequency parameter showed a median reduction of 57% (*P* < 0.01) at 6-week follow-up and 67.8% at 12-month follow-up, compared to baseline values [34].

Another study in 2011 sought to retrospectively examine outcomes in 221 gastroparesis patients who underwent Enterra therapy. The subjects’ gastroparesis etiologies were diabetic, idiopathic, or postsurgical. The findings included significant reduction (*P* < 0.05) in the patients’ total symptom scores, hospitalizations, and use of medications (such as prokinetics and antiemetics). The investigators noted greater total symptom score reductions in patients with diabetic gastroparesis and postsurgical gastroparesis, when compared to idiopathic gastroparetics. All groups manifested a significant weight increase. The number of patients with J-tube decreased after GES implantation. Importantly, follow-up gastric emptying tests for the patients revealed “similar, abnormal delays in mean gastric retention” compared to baseline [35].

In a prospective, multicenter crossover study focusing exclusively on idiopathic gastroparesis patients, McCallum et al. set out to assess the effects of having the Enterra stimulator device turned “ON” for the initial 1.5 months, followed by double-blinded and randomized placement into either the “ON” or “OFF” groups for the crossover stage. Ultimately, the collaborators noted significant reduction in weekly vomiting frequency during the unblinded “ON” period (61.2%, *P* < 0.001) along with a non-significant reduction (17%) in the subsequent “ON” vs. “OFF” stages. At the 1-year follow-up in patients with consecutive “ON” stimulation, there was a continued decrease in vomiting and length of hospitalizations for these patients [36].

Furthermore, a single-center prospective study analyzed the safety and efficacy of Enterra therapy in 151 patients with refractory gastroparesis. The etiologies of the gastroparesis were either diabetic (48%), idiopathic (48%), or other (4%). At assessment during follow-up (17 ± 11 months after stimulator placement), 75% of the subjects communicated symptomatic improvement. Diabetic patients appeared to show greater improvement compared to their non-diabetic counterparts. Of note, the three symptoms of nausea, anorexia, and early satiety showed the most improvement [37].

Shada et al. assessed the outcomes of 119 gastroparesis patients who underwent Enterra device implantation and medical therapy from 2005 to 2017. Gastroparesis symptoms were improved during follow-up evaluation and a reduction in prokinetic and narcotic medication was also documented. Gastric emptying studies were not routinely administered to patients at their follow-up encounters [38].

Recently, a study from France assessed 172 patients with refractory vomiting who underwent Enterra device implantation. After the device was turned “OFF,” subjects were blindly randomized to an “ON” or “OFF” group. At 4 months, subjects were then crossed over with follow-up assessments conducted at 5 months and 9 months after implantation. A significant reduction in vomiting frequency was observed during the “ON” phase. This finding was echoed in both the diabetic and non-diabetic patients. Furthermore, no reductions in gastric emptying were observed with patients receiving GES [39].

It is necessary to highlight that GES via Enterra therapy has not been conclusively established to improve gastric emptying. Thus, other avenues through which the Enterra gastric electrical stimulator achieves neuromodulation were reviewed by Yin et al. On a cellular level, one study disclosed that while the degree of ICC reduction did not correspond with the severity of gastroparesis, those subjects with greater ICC depletion levels displayed a lessened symptomatic response to Enterra therapy [40, 41]. On an organ level, Enterra therapy has shown to improve gastric accommodation, “defined as the reflex-mediated postprandial augmentation of gastric volume” [40] among gastroparesis patients. In addition to increased gastric accommodation (measured via gastric barostat), subjects also had a significant reduction in total symptom score [42]. To investigate the effect of GES on gastric slow waves, some researchers subjected gastroparesis patients to 5 days of temporary endoscopically placed GES and found changes in the frequency and amplitude of their slow waves. However, the results were not statistically significant and detailed data is unpublished [40]. Another potential mechanism of Enterra therapy is via increased vagus nerve efferent autonomic function. One study posited that the significant symptomatic improvement in patients undergoing GES may be attributed to neurostimulation ascending from the stomach to the brain via vagal afferents, which may influence the nausea and vomiting centers of the brain [42].

Here, it is important to mention there are several factors which predict response to the treatment with Enterra. The presence of abdominal pain; narcotic use; and comorbidities such as migraine headaches, anorexia, bulimia, rumination syndrome, cyclic vomiting syndrome, and marked dysrhythmia (tachygastria) are associated with poor outcome. However, diabetes is associated with a better response to gastric electrical stimulation [43••]. As discussed in the “Pathophysiology of Gastroparesis” section, nausea responds better to GES compared to other symptoms. Therefore, when nausea is the dominant symptom, a better response to GES would be predicted [43••].

## Gastric Electrical Neurostimulation Combined with Pyloroplasty

A pervading theme throughout much of the motility literature is the inconclusive and unpredictable findings on the impact of GES on gastric emptying. Several studies have reported that GES did not decrease gastric transit in gastroparesis patients [35, 39]. In a prospective study, patients who underwent GES in conjunction with Heineke-Mikulicz pyloroplasty showed significant improvements in total symptom score and gastric emptying studies, in comparison to their counterparts who only underwent GES. Symptom improvement was 45% (*P* < 0.001) in the former group, compared to 35% (*P* < 0.001) in the latter. And gastric emptying improved by 45% (*P* < 0.001) at 2 h and 64% (*P* < 0.001) at 4 h in the former group, compared to 13% (non-significant) and 7% (non-significant) respectively in the latter. The authors emphasized that post-vagotomy gastroparesis patients who underwent the two combined interventions exhibit the most significant response with regard to symptom severity, symptom frequency, and gastric emptying results. It is conceivable that GES plus pyloroplasty has the potential to achieve even greater long-term symptomatic control than with GES alone, while improving delayed gastric transit [44].

A subsequent study, in which all subjects underwent simultaneous dual GES device implantation and surgical pyloroplasty, demonstrated significant improvements in total symptoms score and gastric emptying when compared to baseline pre-procedural calculations. The researchers asserted that such improvements also helped in reducing the length of hospitalizations for these patients and in combating malnutrition, as evidenced by increased weight gain observed during follow-up [45••].

## Complications

GES system complications can generally be classified into the following categories: dislodgement of GES electrodes, penetration of electrodes through the gastric mucosa, lead insulation damage, erosion or migration of the lead or neurostimulator, and bowel obstruction [31]. One particular concern for GES patients is an infection at the pulse generator site. Precipitating events can include trauma, injury, or falls. Given that a large subset of GES patients have diabetic gastroparesis, these individuals can be predisposed to developing infections due to their microvasculature pathology and compromised immune system. Along with infection, other indications for removal of the GES pulse generator from patients include lack of symptomatic improvement, lead dislodgement, small bowel obstruction, peptic ulcer, penetration of the electrodes through the gastric mucosa, lead insulation damage, erosion or migration of the lead or neurostimulator, repositioning of the GES system due to trauma or twisted wires, or migration of the device [31].

## Conclusion

Gastric electrical neurostimulation has shown to be a viable option of managing refractory gastroparesis with significant improvements in multiple cardinal symptoms including nausea and vomiting. Objective improvement in gastric emptying with GES has generally been lacking in trials. Such observation prompted the addition of surgical pyloroplasty to GES and results thus far have revealed an augmentation of the symptomatic improvement seen by GES therapy alone. Furthermore, GES coupled with pyloroplasty significantly decreases gastric transit time as compared to baseline. While the results are promising, large-scale controlled and randomized studies are required in the future to fully elucidate the effects, complications, and determinants of response.
